# Multimodal PCSC Sensors for Real-Time Temperature and Force Detection Using LRTNet

**DOI:** 10.3390/s26113506

**Published:** 2026-06-02

**Authors:** Zhiqiang Gao, Bing Ren, Jing Han, Jie Li, Jing Liu, Huihui Bai

**Affiliations:** 1 Department of Automation, Taiyuan Institute of Technology, Taiyuan 030008, China; gaozhiqiang@tit.edu.cn (Z.G.); baihuihui@tit.edu.cn (H.B.); 2 Department of Public Security Administration, Shanxi Police College, Taiyuan 030051, China; renexcellent@163.com; 3 College of Mechatronic Engineering, North University of China, Taiyuan 030051, China; 4 College of Materials Science and Engineering, North University of China, Taiyuan 030051, China; ajiestar@nuc.edu.cn (J.L.); b2020503015@st.nuc.edu.cn (J.L.)

**Keywords:** multimodal sensors, signal crosstalk, real-time PCSC sensor, LRTNet

## Abstract

Multimodal sensors can collect multiple signals and have great potential in robotics and other technical fields. However, such sensors often encounter challenges of signal crosstalk and insufficient real-time performance, particularly in the detection of pressure and temperature, which significantly affect measurement accuracy. To address this issue, a multimodal PCSC sensor was developed. This sensor reduces signal crosstalk by separating force and temperature signals. It uses the pressure-resistance variation of carbon quantum dots (CQDs) to detect force and the thermochromic properties of spiropyran (SP) to detect temperature. When pressure and temperature act on the sensor simultaneously, the resistance increases with pressure and stabilizes when the pressure becomes constant. The response time is 0.4 s. As the temperature rises, the resistance decreases, and the color becomes deeper. Both resistance and color stabilize within 7.5 s. To improve temperature sensing accuracy, a lightweight ResNet-Transformer network (LRTNet) was proposed. This algorithm combines ResNet’s ability to extract features and Transformer’s ability to model sequences. It efficiently fuses color and resistance signals for temperature detection. Tests on a robotic manipulator for dual recognition of temperature and force showed that LRTNet achieved a runtime of 152.08 ms and a temperature sensing accuracy of 95%. LRTNet improved overall performance by at least 11% compared to traditional algorithms. The sensor and algorithm improved the performance and reliability of multimodal sensors.

## 1. Introduction

In fields such as industrial control, healthcare, and environmental monitoring, precise multimodal sensing technology is crucial for enhancing system performance. However, multimodal sensors encounter challenges such as signal crosstalk and limited real-time performance, which significantly restrict measurement accuracy and system stability [1,2,3]. As a result, developing high-performance sensors capable of independently sensing multiple physical quantities, along with effective multimodal fusion algorithms, has become a key focus of current research.

When multimodal sensors simultaneously sense force and temperature, signal crosstalk often occurs, resulting in interference between force and temperature signals. This interference complicates the accurate differentiation of changes in physical quantities, thereby reducing sensing accuracy [4,5]. To address this issue, researchers have proposed three primary solutions: multilayer structural design, frequency-selective decoupling, and functional material separation mechanisms. The first approach, multilayer structural design, reduces signal crosstalk by distributing the force and temperature sensing functions across distinct material layers [6,7]. For example, a multimodal flexible sensor developed by Zhou et al. employs a multilayer structure to independently sense pressure and temperature, achieving high-sensitivity pressure detection and a linear temperature response [8]. Despite this design’s ability to reduce interference by physically separating signal paths, the coupling effect between layers may still cause signal distortion during repeated bending or stretching, while the multilayer design itself increases the sensor’s thickness and manufacturing complexity. The second approach, frequency-selective decoupling, achieves signal separation by leveraging different material responses at various frequencies, allowing force and temperature signals to be transmitted in distinct frequency ranges and thus avoiding interference [9,10,11,12,13]. While this method enhances detection accuracy and is particularly suitable for systems that transmit signals via changes in capacitance or resistance, it also requires complex electronic circuits to precisely control signals, and certain materials exhibit unstable responses at high frequencies, limiting its applicability. The third approach, functional material separation mechanisms, involves selecting materials with unique physical response characteristics to isolate force and temperature signals at the material level [14,15]. For instance, Han et al. developed a thermosensitive conductive hydrogel that detects temperature within the range of 10 °C to 75 °C, while resistance changes are used to independently detect force signals [16]. Although effective in reducing signal crosstalk, this mechanism imposes high requirements on material selection and stability. Recently, thermochromic materials have presented promising solutions to signal crosstalk issues. Materials such as spiropyran sense temperature through color changes, while force signals are detected via resistance changes. Since these two mechanisms operate based on different physical principles, they function without mutual interference, significantly improving detection accuracy and reliability [17,18]. This approach has proven to be an effective solution for signal decoupling in multimodal sensors.

In the development of multimodal fusion algorithms, researchers have explored various methods, including data-level fusion, feature-level fusion, model-level fusion, and decision-level fusion [19,20,21,22,23]. Each method has distinct advantages, limitations, and applications [24,25,26]. For example, data-level fusion integrates raw data from multimodal sensors, which is suitable for cases where the data relationships are direct and the data volume is moderate. Heon Ick Park employed data-level fusion using flexible thermoelectric devices (TED) and resistive temperature detectors (RTD) via a one-dimensional convolutional neural network (1D-CNN), achieving an 88% recognition rate for ten objects with varying temperatures [27]. Despite its simplicity, this method may lack flexibility when managing complex relationships. Feature-level fusion combines data features from different modalities to form a comprehensive feature representation, enhancing the model’s learning capacity. For instance, Zhao integrated temperature and pressure data from flexible sensors using a convolutional neural network (CNN) algorithm for feature fusion, achieving an object recognition accuracy of 99% [28]. This method is suitable when data preprocessing and feature extraction are standardized, though it relies heavily on effective feature extraction algorithms and is limited by computational resources. Model-level fusion integrates data at intermediate layers of the model, with each modality undergoing independent processing prior to fusion. This approach is effective for handling complex, diverse data but demands higher computational resources [29]. Lastly, decision-level fusion combines predictions from modality-specific models at the decision stage, making it appropriate for scenarios where multimodal data characteristics vary significantly and computational resources are sufficient. While these fusion algorithms have shown potential in enhancing system performance, challenges such as high computational complexity and limited real-time performance remain, especially in resource-constrained environments.

In the previous discussion, the signal crosstalk issues faced by multimodal sensors primarily arise from mutual interference among multiple signals, leading to a decrease in sensing accuracy. The problem of poor real-time performance is often due to the high computational complexity of signal decoupling methods or data fusion algorithms, which in turn impacts system response speed. To address these challenges, a PCSC sensor capable of separating force and temperature signals is introduced, along with the design of a LRTNet to optimize the data fusion process. The PCSC sensor leverages the thermochromic properties of SP and the pressure-resistance response of CQDs to achieve sensing capabilities, detecting temperature and pressure signals through changes in color and resistance, respectively. Meanwhile, LRTNet integrates ResNet’s feature extraction capabilities with Transformer’s temporal modeling, enabling efficient processing of limited sample data to enhance the real-time performance and accuracy of the overall sensing system.

The structure of this paper is as follows: Section 2 details the preparation method for the PCSC sensor and the development of the fusion algorithm. Section 3 describes the experimental setup, presents the experimental results, and compares these results with existing technologies. Section 4 validates the algorithm’s effectiveness in robotic force and temperature dual-recognition experiments. Finally, Section 5 summarizes the findings and discusses potential directions for future research.

## 2. Methodology

### 2.1. Overview of the Research Methodology

**Figure 1:**
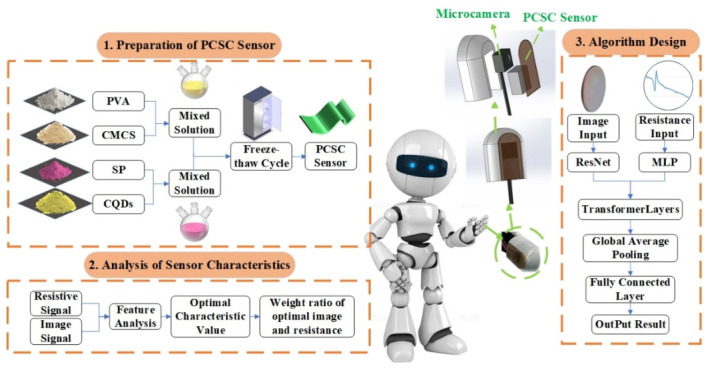
The overall architecture.

An innovative approach based on multimodal smart hydrogels is proposed, involving the development of a sensor fusion algorithm that integrates color recognition and temperature sensing. As shown in Figure 1, the methodology comprises three key steps: First, the PCSC sensor is fabricated using polyvinyl alcohol (PVA), carboxymethyl chitosan (CMCS), SP, and CQDs, forming a dual-state hydrogel sensor with thermochromic and resistance-changing properties. Next, sensor characteristics are analyzed, with experimental setups used to collect resistance and image signals, examining the sensor’s response under varying temperatures. Finally, a fusion algorithm is developed, combining lightweight ResNet and MLP models to process both image and resistance signals. Feature fusion is performed through the Transformer layer, with classification achieved via a fully connected layer, enabling high real-time performance and accuracy in temperature recognition.

### 2.2. Preparation of the Sensor

A dual-responsive polyvinyl alcohol double-network hydrogel, PCSC, was designed. This sensor employs biodegradable, water-soluble PVA as the primary network and CMCS as the secondary network, with both forming a double-network hydrogel framework through physical cross-linking via hydrogen bonds. SP functions as the thermochromic filler, while CQDs serve as conductive fillers with optoelectronic properties. The CQDs are uniformly dispersed in the double-network matrix through hydrogen bonding, enhancing the thermochromic effect of SP and providing the hydrogel with high sensitivity to electrical signals. The sources and purity information of the relevant materials are shown in Table 1.

**Figure 2:**
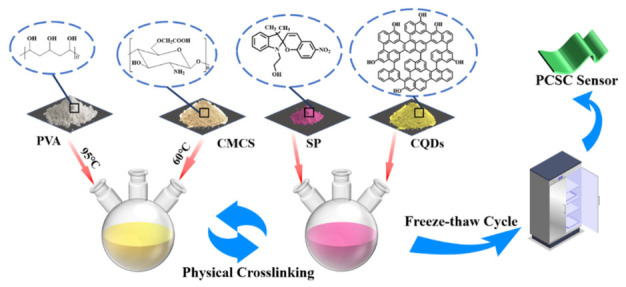
Preparation process of the PCSC hydrogel.

The preparation of the multi-responsive PCSC hydrogel, as shown in Figure 2, involves the following steps:

- Preparation of CQD Powder: The synthesis method of carbon quantum dots in this work was adapted from relevant literature with appropriate optimization and modification [30,31]. A 40 mL sodium hydroxide solution (1.5 mol/L) is gradually added to 40 mL of acetaldehyde and stirred at room temperature for 2 h. The reaction product undergoes ultrasonic treatment for 30 min, is then neutralized with dilute hydrochloric acid, filtered, and washed three times with deionized water. Finally, the product is dried at 70 °C for 8 h to yield CQD powder.
- Preparation of PCSC Hydrogel: 10 g of PVA is dissolved in 80 mL of deionized water and stirred at 95 °C for 1 h to obtain a clear PVA solution, which is left to stand for defoaming. Separately, 2.5 g of CMCS is dissolved in 20 mL of deionized water, stirred at 60 °C for 30 min, and added to the PVA solution to create a PVA/CMCS mixture, which is concentrated to 40 mL by continuous stirring at 60 °C. Next, 3 mg of SP and 75 mg of CQDs are dissolved separately in 10 mL of anhydrous ethanol and subjected to ultrasound for 10 min, resulting in an SP/CQD solution at a ratio of 3:75. Then, 20 mL of the PVA/CMCS mixture is combined with the SP/CQD solution in varying mass ratios. The resulting composite hydrogel solution is stirred at room temperature for 1 h. After degassing, 2.5 mL of the composite solution is injected into a mold using a plastic syringe. The hydrogel undergoes three freeze–thaw cycles by alternately freezing at −30 °C and thawing at room temperature, ultimately forming the PCSC hydrogel samples. In this work, the PCSC hydrogel was prepared based on the method reported for PCS hydrogel, with the additional introduction of carbon quantum dots (CQDs) as a modified composite hydrogel [32,33].

### 2.3. Algorithm Design

**Figure 3:**
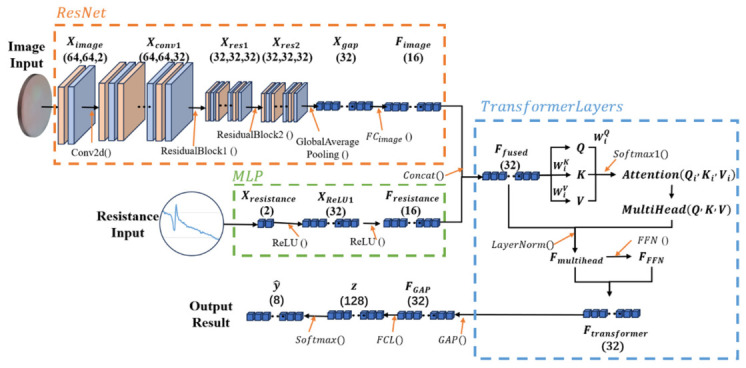
The LRTNet algorithm flowchart.

For dual recognition of force and temperature in small-sample scenarios, a lightweight model is required to efficiently process multimodal data. As shown in Figure 3, image features are extracted using a lightweight ResNet, while resistance features are extracted through an MLP. The Transformer’s self-attention mechanism is then applied to fuse these modal features, effectively capturing correlations between image and resistance data. Global average pooling is subsequently employed to reduce feature dimensions, followed by a fully connected layer for further processing. Finally, the Softmax activation function generates the classification results.

#### 2.3.1. Image Feature Extraction–Lightweight ResNet

The lightweight ResNet model is employed for image feature extraction, specifically designed to reduce computational load and minimize parameters, resulting in a more compact model. By incorporating skip connections, ResNet addresses the vanishing gradient problem often encountered in deep network training, allowing it to effectively capture rich image features even with small-sample data. Its structure is as follows:

(1) $$F_{image}=ResNet(X_{image})$$

a. Input Convolutional Layer: The input image, $X_{image}$, first passes through a convolutional layer (Conv1) to extract preliminary features.

(2) $$X_{conv1}=Conc2d\left(X_{image},W_{conv1},b_{conv1}\right)$$

In this process, $W_{conv1}\;$ and $b_{conv1}$ refer to the convolutional kernel weights and bias, respectively.

b. Residual Block: The residual block is the key module in ResNet, designed to mitigate the vanishing gradient problem in deep networks. Each residual block consists of two convolutional layers, followed by batch normalization (BatchNorm) and ReLU activation.

$$X_{res1}=ReLU(BatchNorm(Conv2d(X_{conv1},W_{res1\_conv1},b_{res1\_conv1})))$$

The residual connection then adds the input to the output:

(3) $$X_{res1}=ReLU(X_{res1_{bn2}}+X_{conv1})$$

The second residual block follows a similar structure.

c. Global Average Pooling Layer: The global average pooling layer reduces the feature map to a scalar, minimizing the number of parameters.

(4) $$X_{gap}=\frac{1}{H\times W}\sum_{i=1}^{H}\sum_{j=1}^{W}X_{res2}\left[i,j,:\right]$$

d. Fully Connected Layer: The pooled features are mapped into a 16-dimensional feature vector.

(5) $$F_{image}=W_{fc}\cdot X_{gap}+b_{fc}$$

Here, $W_{fc}$ and $b_{fc}$ represent the weights and bias of the fully connected layer, respectively.

#### 2.3.2. Feature Fusion Layer

For multimodal data fusion, the Transformer effectively handles sequential data, making it suitable for integrating information from different modalities, such as image and sensor data, thereby enhancing overall model performance. The Transformer’s self-attention mechanism captures long-range dependencies within the input data, which improves the model’s ability to interpret the global data structure. This is particularly beneficial in small-sample scenarios, where generalization capability is critical.

In the feature fusion layer, image features extracted from ResNet and resistance data features are reduced to the same dimensionality through fully connected layers before being fused. The fusion formula is as follows:

(6) $$F_{fused}=\alpha F_{image}+{(1-\alpha )F}_{resistance}$$

Here, *α* represents the fusion weight, determining the proportion of image and temperature features in the fused representation.

The fused features $F_{fused}$ are then processed through the Transformer module. The Transformer captures global dependencies within the data using a multi-head self-attention mechanism, where the attention calculation for each head is defined as:

(7) $$Attention(Q_{i},K_{i},V_{i})=Softmax1(\frac{Q_{i}K_{i}^{T}}{\sqrt{d_{k}}})V_{i}$$

In this equation, $Q_{i}$, $K_{i}$, and $V_{i}$ represent the query, key, and value matrices, respectively. Results from multiple attention heads are combined through a linear transformation, and then further processed by layer normalization and a feedforward neural network.

After passing through the Transformer, the fused features undergo global average pooling to reduce dimensionality, followed by additional processing through fully connected layers. Finally, the classification output is generated via the Softmax activation function.

In the LRTNet design, the lightweight ResNet minimizes model parameters and computational overhead while maintaining efficient image feature extraction. By utilizing the Transformer’s self-attention mechanism, the feature fusion layer effectively integrates multimodal data, significantly enhancing model performance in small-sample conditions. This design not only meets the resource constraints of embedded devices but also ensures high accuracy in force and temperature recognition for multimodal tasks.

## 3. Experimental Design

### 3.1. Sensor Performance Testing

**Figure 4:**
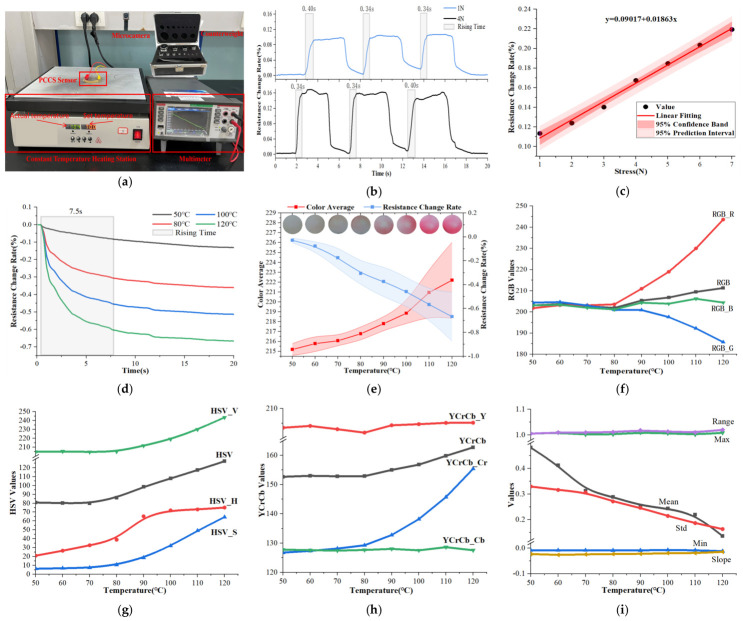
Performance Testing Experiment: (**a**) test setup; (**b**) sensor resistance changes under applied pressure; (**c**) relationship between pressure and sensor resistance; (**d**) sensor color changes with temperature; (**e**) relationship between temperature and sensor color/resistance; (**f**) RGB channel at various temperatures; (**g**) HSV channel at various temperatures; (**h**) YCrCb channel at various temperatures; (**i**) variation curves of six characteristic values of the resistance change rate at different temperatures.

To evaluate the performance of the PCSC sensor, the test setup shown in Figure 4a was used to record the sensor’s resistance and color changes under varying force and temperature conditions. The setup includes a heating plate, multimeter, micro camera, and weights. The heating platform can accurately maintain the set temperature with a control accuracy of ±1 °C, providing stable and repeatable temperature conditions for the temperature response test. The weights simulate force magnitudes ranging from 1 N to 7 N, while the heating plate simulates temperatures from 50 °C to 120 °C. The multimeter measures resistance changes, and the camera captures color changes in the sensor.

Mechanical performance testing was conducted initially. Forces from 1 N to 7 N were applied sequentially to the sensor, and the corresponding resistance changes were recorded as Figure 4b. It can be observed that the curves are highly consistent in shape, response time and amplitude, with no obvious hysteresis or baseline drift, demonstrating the excellent repeatability of the sensor under pressure loading. Due to variability in initial sensor resistance, the resistance change rate was used to represent the relative change, calculated as follows:

(8) $$Resistance\;change\;rate=\left(R-R_{0}\right)/R_{0}$$

where *R*_0_ is the initial resistance, and *R* is the resistance after force application. Results indicate stable resistance changes under force, with a resistance rise time of approximately 0.4 s when weights are reapplied.

Figure 4c shows the relationship between the applied pressure and the resistance change rate of the sensor. It can be seen that the resistance change rate of the sensor exhibits a good linear relationship with the applied pressure. The pressure response sensitivity of the sensor is 1.863 × 10^−2^%/N, indicating that the sensor possesses distinct and stable pressure sensitivity. Regarding the pressure accuracy of the sensor, the 95% prediction interval in Figure 4c covers all test data points, demonstrating that the pressure-resistance relationship model has favorable prediction accuracy and can be used for the quantitative detection of pressure.

Next, temperature performance testing was performed. When the heating plate reached the set temperature, the sensor was placed on it for 15 s, during which resistance changes were recorded by the multimeter and color changes were captured by the camera. As shown in Figure 4d, Within the temperature range of 50–120 °C, the resistance change rate of the sensor increases significantly with rising temperature, showing a clear temperature-dependent response trend. And resistance stabilized around 7.5 s, contrasting with a 0.4 s stabilization time under force. This variation in response times effectively distinguishes force from temperature responses, avoiding signal crosstalk.

Figure 4e illustrates the effect of rising temperatures on the sensor’s color and resistance. As the temperature increased from 50 °C to 120 °C, the sensor color shifted from light gray to deep red, with the average color value (red curve) decreasing from 228 at 50 °C to 215 at 120 °C, indicating noticeable darkening. Simultaneously, the resistance change rate (blue curve) declined from 0 at 50 °C to −0.8 at 120 °C, showing a decrease in resistance with rising temperature. Notably, higher temperatures correlate with a greater absolute resistance change rate, approximately −0.4 at 80 °C and close to −0.8 at 120 °C. Shaded areas in the figure represent measurement errors, which are more pronounced at higher temperatures, possibly due to material stability limitations under elevated conditions.

In summary, the PCSC sensor demonstrates high sensitivity in temperature detection, accurately capturing the dual influence of temperature on both color and resistance. Furthermore, it effectively differentiates between force and temperature in recognition tasks.

### 3.2. Algorithm Testing and Optimization

#### 3.2.1. Data Collection and Feature Analysis

As shown in Figure 4b,d, during force detection, the sensor’s resistance stabilizes within 0.4 s and exhibits a linear relationship with applied force, while no significant changes are observed in the image data. Therefore, for pressure recognition, the image weight is set to 0, and the resistance weight is set to 10. Conversely, during temperature detection, the changes in the sensor’s color and resistance are nonlinear, necessitating the design and optimization of a temperature-focused algorithm.

In this experiment, data samples were collected across eight temperatures: 50 °C, 60 °C, 70 °C, 80 °C, 90 °C, 100 °C, 110 °C, and 120 °C. At each temperature, 200 samples were gathered, consisting of 100 image samples and 100 resistance samples. To improve algorithm efficiency, the color characteristics of the RGB, HSV, and YCrCb channels, along with six characteristic values of the resistance change rate (range, max, min, std, mean, slope), were analyzed to select optimal features. As shown in Figure 4f–h, between 80 °C and 120 °C, the S value in the HSV channel demonstrated the fastest rate of change, providing clear temperature distinctions. Between 50 °C and 80 °C, changes in the RGB and YCrCb channels were relatively slower, but the H value in the HSV channel showed a more pronounced rate of change. Figure 4i shows the curves of six characteristic values of the resistance change rate at different temperatures. It can be seen that the mean and standard deviation exhibit the most significant changes.

To further refine data analysis, the average rate of change (ARC) in each range was calculated using the following formula:

(9) $$Y=\frac{1}{n-1}\sum_{i=1}^{n-1}\frac{x_{i+1}-x_{i}}{x_{i}}$$

Table 2 presents the average rates of change (ARC) for various feature values across the 50 °C to 120 °C range, which informed the selection of the most suitable feature values. In the 50 °C to 80 °C range, HSV_H exhibited the highest ARC at 22.94%, while in the 80 °C to 120 °C range, HSV_S had the highest ARC at 57.64%. Within the RGB channel, the R value showed a change rate of 4.60% between 80 °C and 120 °C, indicating a degree of distinction. For resistance features, std and mean displayed the largest change rates at −15.76% and −9.46%, respectively. Regarding color changes, the transition primarily involved a shift from white to pink, influencing hue (H) and saturation (S), while brightness (V) remained relatively constant. Thus, HSV_H and HSV_S were selected as key features to enhance temperature recognition accuracy and real-time performance. For resistance characteristics, std and mean were chosen as primary features to improve precision in temperature detection.

#### 3.2.2. Selection of Weight Ratios for Image and Resistance Data

After identifying the optimal feature values, determining the weight ratio between image and resistance (I:R) data is crucial for optimizing the algorithm’s accuracy and recognition time. To assess the impact of varying weight ratios, the experiment evaluated 11 configurations: 0:10, 1:9, 2:8, 3:7, 4:6, 5:5, 6:4, 7:3, 8:2, 9:1, and 10:0. The accuracy and recognition time under each weight configuration were analyzed to determine the optimal balance.

**Figure 5:**
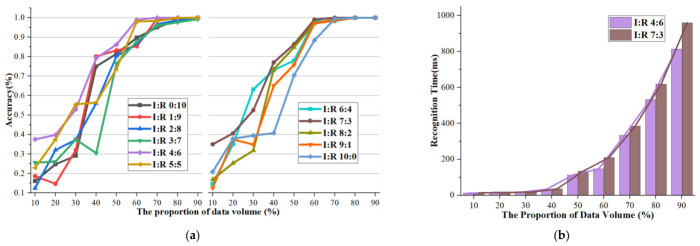
(**a**) Algorithm accuracy across different weight ratios; (**b**) algorithm recognition time across different weight ratios.

As shown in Figure 5a, the weight ratio has a pronounced impact on algorithmic performance. When I:R is set to 4:6 or 7:3, the algorithm achieves an effective balance, allowing both data sources to synergistically improve recognition performance. Even with a limited sample size, these two ratios yield relatively high recognition accuracy. For example, with a training set comprising only 10% of available samples, recognition accuracy exceeds 35%. When the training set reaches 50%, accuracy increases to over 86%. In contrast, when the algorithm relies solely on either image or resistance data, recognition performance declines, indicating that a single data source does not provide sufficient information for robust performance. The combined use of image and resistance data significantly enhances recognition accuracy, underscoring the importance of multimodal data fusion.

Figure 5b illustrates that algorithmic runtime increases with a higher proportion of image data, as image processing typically requires greater computational resources and time. For instance, with a 50% training set, the I:R ratio of 4:6 results in a runtime of 112.45 ms, whereas the 7:3 ratio yields a runtime of 134.04 ms. When the training set is increased to 80%, the recognition time for the 4:6 ratio is 533.41 ms, compared to 618.48 ms for the 7:3 ratio. Consequently, to balance recognition accuracy and computational efficiency, the final weight ratio for image and resistance data was set to 4:6. This allocation ensures high recognition accuracy under low-sample conditions while maintaining a reasonable processing time.

#### 3.2.3. Algorithm Comparison

To evaluate the performance of various algorithms with an I:R ratio set at 4:6, this study compares five algorithms—MLP, LSTM, ResNet16, Transformer, and LRTNet—across different data volumes. The comparison metrics include real-time performance, accuracy, precision, recall, and F1-score, providing a comprehensive evaluation of each algorithm’s strengths and limitations in processing multimodal data, and aiding in selecting the most suitable algorithm.

**Figure 6:**
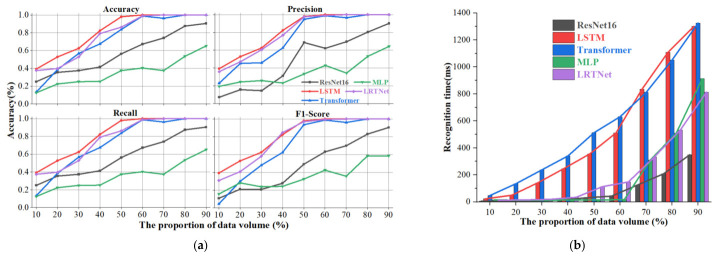
(**a**) Algorithm performance comparison; (**b**) recognition time comparison.

To investigate the effect of data volume on algorithm performance, we conducted multiple sets of experiments with training set ratios ranging from 10% to 90%. The remaining data (90% to 10%) was used as an independent test set, with no sample overlap between the training and test sets, thus avoiding data leakage. As shown in Figure 6a, when data volume exceeds 50%, LSTM, Transformer, and LRTNet achieve over 80% in accuracy, precision, recall, and F1-score, with LSTM surpassing 90% across all four metrics, indicating the best performance. This can be attributed to LSTM’s effectiveness in handling time series and capturing long-term dependencies, making it particularly suitable for larger datasets and complex patterns. Across the 10–90% data range, LSTM and LRTNet exhibit the highest performance, whereas ResNet16 and MLP display relatively lower performance.

Figure 6b illustrates that as data volume increases, identification time for all algorithms also rises. ResNet16 demonstrates the shortest identification time across all data volumes, with runtimes of 29.37 ms at 50% data volume and 208.61 ms at 80%. This efficiency is attributed to ResNet’s residual network structure, which reduces computational load and accelerates processing. MLP and LRTNet follow, with identification times of 14.76 ms and 112.45 ms at 50% of the training set and 508.05 ms and 533.41 ms at 80%, respectively. MLP’s simplicity contributes to its computational speed, while LRTNet’s lightweight design balances efficient processing with high recognition accuracy.

To comprehensively evaluate each algorithm’s performance, an integrated performance metric was introduced, combining accuracy, precision, recall, F1-score, and runtime. The composite indicator is calculated as follows:

(10) $$\begin{array}{c} Composite\;indicator\\ \;\;\;\;\;=\omega_{1}\frac{Accurecy}{100}+\omega_{2}\frac{Precision}{100}+\omega_{3}\frac{Recall}{100}+\omega_{4}\frac{F1-Score}{100}\\ \;\;\;\;\;-\omega_{5}\frac{Time}{max(Time)} \end{array}$$

Here, $\omega_{1}$ to $\omega_{5}$ represent the weights assigned to each performance metric, with $\omega_{1}+\omega_{2}+\omega_{3}+\omega_{4}+\omega_{5}=1.$ The weights of the comprehensive performance metric are set as follows: $\omega_{1}$ (Accuracy) = 0.25, $\omega_{2}$ (Precision) = 0.20, $\omega_{3}$ (Recall) = 0.20, $\omega_{4}$ (F1-score) = 0.25, $\omega_{5}$ (Runtime) = 0.10. First, accuracy directly reflects the overall recognition performance of the algorithm. As the harmonic mean of precision and recall, the F1-score effectively balances the recognition reliability and target detection rate of the model. Together, they constitute the core indicators for evaluating recognition capability, so both are assigned the highest weight of 0.25. Second, consideration is given to both the reliability and completeness of classification results: precision measures the credibility of recognition outcomes to avoid false positives, while recall measures the recognition coverage of target samples to avoid false negatives. As indispensable key indicators for evaluating multi-classification task performance, they play equally important roles in ensuring the validity of recognition results, and are thus each assigned a weight of 0.20. Finally, runtime serves as a critical constraint for the practical deployment of sensors and is assigned a weight of 0.10. This ensures that recognition accuracy is achieved while taking into account the computational efficiency of the algorithm, preventing the exclusive pursuit of recognition performance at the expense of practical application feasibility. The runtime metric is normalized and then inverted to reflect its negative impact on overall performance. Table 3 shows a comparison of the comprehensive performance metrics for different algorithms.

According to Table 3, LRTNet achieved the best performance across all data volumes, peaking at 0.75 with 60% of the data, underscoring its advantages in both accuracy and real-time processing. LSTM showed strong results with lower data volumes, as its comprehensive performance metric rose from 0.21 to 0.60, though a slight decline was noted at the 70% data level. ResNet16 demonstrated consistent performance, with the metric increasing from 0.11 to 0.67. Although the Transformer model performed less optimally at lower data volumes, it steadily improved as data volume increased, reaching a metric value of 0.60 at 90% data. MLP displayed relatively lower performance across all data volumes, with a peak value of 0.37.

Overall, LRTNet demonstrates exceptional performance in terms of accuracy, precision, recall, and F1-score, while maintaining moderate recognition times. This balance makes it particularly suitable for applications requiring high responsiveness and accuracy in low-sample conditions. LRTNet’s lightweight structure and efficient feature extraction capabilities enable it to achieve high accuracy and process real-time data efficiently.

## 4. Real-Time Recognition Experiment on Robotic Gripper

**Figure 7:**
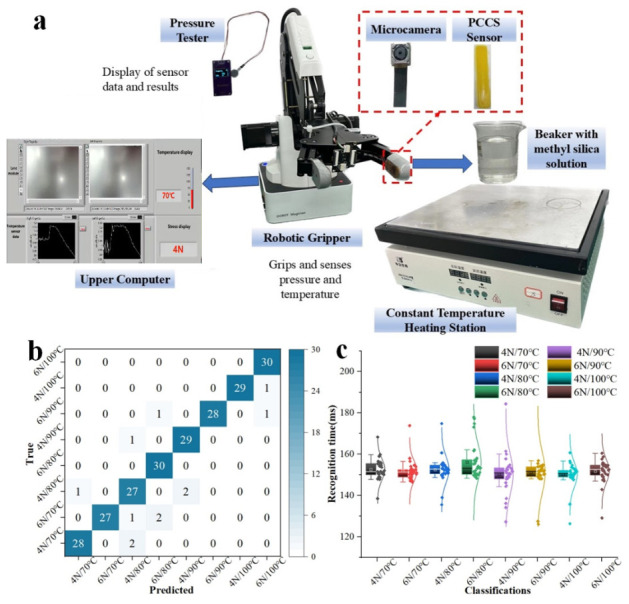
Robotic Gripper Testing Experiment: (**a**) test setup; (**b**) confusion matrix of recognition; (**c**) box plot of recognition times.

An experiment was conducted to validate the performance of the selected algorithm in recognizing beaker temperature and pressure using a robotic gripper. As illustrated in Figure 7a, the experimental setup includes a heating platform for the beaker, a robotic gripper, a beaker, a LABVIEW-based host computer, a micro camera, the PCSC sensor, and a pressure tester. The micro camera and PCSC sensor are integrated into the robotic fingers, while the LABVIEW platform displays the sensor’s resistance variations, real-time footage from the camera, and the final temperature and pressure recognition results. To confirm the accuracy of the robotic gripper’s pressure recognition, the pressure tester measured the actual force applied to the beaker under different conditions.

Building on the algorithm selection and performance testing described above, the LRTNet algorithm was employed for real-time recognition tests across various temperature and pressure combinations. Specifically, classification performance was examined under pressures of 4 N and 6 N at temperatures of 70 °C, 80 °C, 90 °C, and 100 °C. The experiment involved 30 trials for each combination, yielding recognition rates and recognition time distributions. As shown in the confusion matrix in Figure 7b, the LRTNet algorithm achieved an average recognition rate exceeding 95%. Recognition accuracy improved notably at temperatures above 80 °C, with recognition rates surpassing 93% for all combinations from 80 °C to 100 °C. At 70 °C, however, some misclassification occurred due to minimal visual feature changes in this lower temperature range, which presented challenges for feature distinction. Figure 7c presents a box plot of recognition times across different temperature and pressure combinations. The data show that at 70 °C and 80 °C, the average recognition times were 152.64 ms and 151.93 ms, respectively, demonstrating similar performance across these temperatures. At 90 °C and 100 °C, recognition times ranged between 150 ms and 155 ms, with an average of 152.08 ms. These results indicate that the LRTNet algorithm maintains stable processing efficiency at higher temperatures, providing a robust real-time performance suitable for temperature and pressure monitoring in robotic applications.

## 5. Conclusions

These multimodal stimulus-response sensors have broad applications in modern sensing technologies, yet there are challenges such as signal crosstalk and limitations in real-time response constrain measurement accuracy. This study presents a multimodal sensor, PCSC, constructed using PVA, CMCS, SP, and CQDs. This combination results in a bistate hydrogel sensor with thermochromic and resistive properties, effectively separating force and temperature signals to mitigate signal crosstalk. Furthermore, a recognition algorithm based on the LRTNet model was developed, integrating ResNet and Transformer architectures. ResNet employs residual connections to address the vanishing gradient problem and preserve deep learning capabilities, while Transformer’s self-attention mechanism captures global features. Within LRTNet, ResNet extracts image features, MLP processes resistance data, and these features are subsequently fused for classification. The lightweight design of LRTNet ensures high accuracy while reducing computational complexity, making it suitable for real-time applications.

Temperature samples were collected across a range of 50 °C to 120 °C, including both image and resistance data. Performance testing and analysis demonstrated LRTNet’s superior capability in integrating image and resistance data. Comparative experiments with five other algorithms, including LSTM, showed that LRTNet outperformed others by at least 11% in overall metrics. In a validation experiment involving robotic force and temperature recognition, LRTNet achieved an average recognition accuracy of 95% and an average recognition time of 152.08 ms, confirming its high accuracy, low computational complexity, and fast processing speed.

Despite these significant achievements, some limitations remain. The sensor’s temperature response rate is relatively slow; future research should focus on improving this response speed while maintaining accuracy. Additionally, further optimization of LRTNet’s computational complexity is necessary to meet the demands of high real-time applications. Advancements in these areas are expected to further enhance the performance of multimodal sensors, providing more effective solutions for intelligent sensing across various fields.

## Figures and Tables

**Table 1:** The sources and purity information of the relevant materials.

Material	Specification	Manufacturer
PVA	87.00–89.00%	Shanghai Aladdin Biochemical Technology Co., Ltd. (Shanghai, China)
CMCS	99%	Shanghai Macklin Biochemical Technology Co., Ltd. (Shanghai, China)
SP	96%	Shanghai Aladdin Biochemical Technology Co., Ltd. (Shanghai, China)
Anhydrous ethanol	99%	Shanghai Aladdin Biochemical Technology Co., Ltd. (Shanghai, China)
Sodium hydroxide	96%	Tianjin Beichen Fangzheng Reagent Factory (Tianjin, China)
Hydrochloric acid	Analytical grade	Tianjin Beichen Fangzheng Reagent Factory (Tianjin, China)
Acetaldehyde	40%	Shanghai Macklin Biochemical Technology Co., Ltd. (Shanghai, China)
Deionized water	——	Self-made in laboratory

**Table 2:** Average Rate of Change (ARC).

Feature	ARC from 50 °C to 120 °C (%)	ARC from 50 °C to 80 °C (%)	ARC from 80 °C to 120 °C (%)
RGB	0.57	0.19	1.14
RGB_R	2.75	0.29	4.60
RGB_G	−1.35	−0.56	−1.95
RGB_B	0.10	−0.32	0.41
HSV	6.83	2.24	10.28
HSV_H	21.61	22.94	20.62
HSV_S	41.93	20.99	57.64
HSV_V	2.54	0.05	4.40
YCrCb	0.92	0.05	1.57
YCrCb_Y	0.12	−0.28	0.42
YCrCb_Cr	2.98	0.65	4.72
YCrCb_Cb	−0.02	−0.03	−0.01
Mean	−15.76	−15.35	−16.06
Std	−9.46	−6.22	−11.88
Min	6.47	2.10	9.75
Max	0.05	−0.09	0.15
Range	0.21	0.19	0.22
Slope	−5.89	0.26	−10.51

**Table 3:** Comprehensive Performance Metric.

Algorithm	The Proportion of Data Volume (%)								
	10%	20%	30%	40%	50%	60%	70%	80%	90%
ResNet16	0.11	0.21	0.21	0.28	0.44	0.51	0.55	0.64	0.67
LSTM	0.21	0.35	0.30	0.51	0.58	0.64	0.60	0.60	0.60
Transformer	−0.09	0.10	0.08	0.32	0.42	0.59	0.58	0.61	0.60
MLP	0.06	0.18	0.18	0.19	0.27	0.33	0.22	0.35	0.37
LRTNet	0.23	0.31	0.42	0.62	0.67	0.75	0.72	0.70	0.68

## Data Availability

The original contributions presented in this study are included in the article/Supplementary Material. Further inquiries can be directed to the corresponding author.

## Supplementary Materials

The following supporting information can be downloaded at: https://www.mdpi.com/article/10.3390/s26113506/s1, Video S1: Recognition Experiment on Robotic Gripper.

## References

[1] Xu S., Fan Z., Yang S., Zuo X., Guo Y., Chen H., Pan L. (2021). Highly Flexible, Stretchable, and Self-Powered Strain-Temperature Dual Sensor Based on Free-Standing PEDOT:PSS/Carbon Nanocoils-Poly(vinyl) Alcohol Films. ACS Sens..

[2] Zhang Q., Liu Z., Wu J., Sun P., Zhang H. (2025). Design and Performance Analysis of a Hybrid Flexible Pressure Sensor with Wide Linearity and High Sensitivity. Sensors.

[3] Yin G., Tian C., Jiang Q., Wang G., Shao L., Li Q., Li Y., Yu M. (2026). Fabrication and Sensing Characterization of Ionic Polymer-Metal Composite Sensors for Human Motion Monitoring. Sensors.

[4] He Y., Xu X., Xiao S., Wu J., Zhou P., Chen L., Liu H. (2024). Research Progress and Application of Multimodal Flexible Sensors for Electronic Skin. ACS Sens..

[5] Guo Y., Wei X., Gao S., Yue W., Li Y., Shen G. (2021). Recent Advances in Carbon Material-Based Multifunctional Sensors and Their Applications in Electronic Skin Systems. Adv. Funct. Mater..

[6] Zhou Z., Liu K., Ban Z., Yuan W. (2022). Highly adhesive, self-healing, anti-freezing and anti-drying organohydrogel with self-power and mechanoluminescence for multifunctional flexible sensor. Compos. Part A Appl. Sci. Manuf..

[7] Hu F., Cheng L., Fan S., Xue X., Liang Y., Lu M., Wang W. (2022). Chip-level orthometric surface acoustic wave device with AlN/metal/Si multilayer structure for sensing strain at high temperature. Sens. Actuators A Phys..

[8] Zhou Z., Guo K., Yin F., Yue W., Li Y., Yin J. (2023). The dual-mode sensing of pressure and temperature based on multilayer structured flexible sensors for intelligent monitoring of human physiological information. Compos. Sci. Technol..

[9] Wu T., Li Y.T., Zhao L., Zhang Y., Zhang Z., Yuan J., Wu Y., Che A., Ma Y., Chai Y. (2026). Recent Progress on Flexible Multimodal Sensors: Decoupling Strategies, Fabrication and Applications. Adv. Mater..

[10] Yin Y., Wang Y., Li H., Xu J., Zhang C., Li X., Cao J., Feng H., Zhu G. (2022). A flexible dual parameter sensor with hierarchical porous structure for fully decoupled pressure–temperature sensing. Chem. Eng. J..

[11] Kose U., Sili G., Doken B., Saygili E.S., Akleman F., Kartal M. (2026). A New Hybrid Sensor Design Based on a Patch Antenna with an Enhanced Sensitivity Using Frequency-Selective Surfaces (FSS) in the Microwave Region for Non-Invasive Glucose Concentration Level Monitoring. Electronics.

[12] Ma Z., Zhang J., Li J., Shi Y., Pan L. (2020). Frequency-Enabled Decouplable Dual-Modal Flexible Pressure and Temperature Sensor. IEEE Electron Device Lett..

[13] Fastier-Wooller J.W., Dau V.T., Dinh T., Tran C.-D., Dao D.V. (2021). Pressure and temperature sensitive e-skin for in situ robotic applications. Mater. Des..

[14] Fei W., Jianwen C., Xihua C., Xining L., Xiaohua C., Yutian Z. (2022). Wearable Ionogel-Based Fibers for Strain Sensors with Ultrawide Linear Response and Temperature Sensors Insensitive to Strain. ACS Appl. Mater. Interfaces.

[15] Chen J., Wen H., Zhang G., Lei F., Feng Q., Liu Y., Cao X., Dong H. (2020). Multifunctional Conductive Hydrogel/Thermochromic Elastomer Hybrid Fibers with a Core-Shell Segmental Configuration for Wearable Strain and Temperature Sensors. ACS Appl. Mater. Interfaces.

[16] Han Y., Liu Y., Liu Y., Jiang D., Wu Z., Jiang B., Yan H., Toktarbay Z. (2025). High-performance PVA-based hydrogels for ultra-sensitive and durable flexible sensors. Adv. Compos. Hybrid Mater..

[17] Lu Y., Qu X., Wang S., Zhao Y., Ren Y., Zhao W., Wang Q., Sun C., Wang W., Dong X. (2021). Ultradurable, freeze-resistant, and healable MXene-based ionic gels for multi-functional electronic skin. Nano Res..

[18] Xiao S., He Y., Lu Y., Niu X., Li Q., Wu J., Luo D., Tian F., Wan G., Liu H. (2023). An ultrasensitive flexible pressure, temperature, and humidity sensor based on structurally adjustable nano-through-hole array films. J. Mater. Chem. C Mater. Opt. Electron. Devices.

[19] Chun S., Kim J.-S., Yoo Y., Choi Y., Jung S.J., Jang D., Lee G., Song K.-I., Nam K.S., Youn I. (2021). An artificial neural tactile sensing system. Nat. Electron..

[20] Wei X., Li H., Yue W., Gao S., Chen Z., Li Y., Shen G. (2022). A high-accuracy, real-time, intelligent material perception system with a machine-learning-motivated pressure-sensitive electronic skin. Matter.

[21] Liu M., Zhang Y., Wang J., Qin N., Yang H., Sun K., Hao J., Shu L., Liu J., Chen Q. (2022). A star-nose-like tactile-olfactory bionic sensing array for robust object recognition in non-visual environments. Nat. Commun..

[22] Duan S., Wang B., Lin Y., Li Y., Zhu D., Wu J., Xia J., Lei W., Wang B. (2021). Waterproof Mechanically Robust Multifunctional Conformal Sensors for Underwater Interactive Human–Machine Interfaces. Adv. Intell. Syst..

[23] Sun Z., Zhu M., Zhang Z., Chen Z., Shi Q., Shan X., Yeow R.C.H., Lee C. (2021). Artificial Intelligence of Things (AIoT) Enabled Virtual Shop Applications Using Self-Powered Sensor Enhanced Soft Robotic Manipulator. Adv. Sci..

[24] Ding Z., Li W., Wang W., Zhao Z., Zhu Y., Hou B., Zhu L., Chen M., Che L. (2024). Highly Sensitive Iontronic Pressure Sensor with Side-by-Side Package Based on Alveoli and Arch Structure. Adv. Sci..

[25] Wu X., Yang X., Wang P., Wang Z., Fan X., Duan W., Yue Y., Xie J., Liu Y. (2024). Strain-Temperature Dual Sensor Based on Deep Learning Strategy for Human–Computer Interaction Systems. ACS Sens..

[26] Duan S., Shi Q., Wu J. (2022). Multimodal Sensors and ML-Based Data Fusion for Advanced Robots. Adv. Intell. Syst..

[27] Ick P.H., Jin C.T., In-Geol C., Suk R.M., Youngsu C. (2023). Object classification system using temperature variation of smart finger device via machine learning. Sens. Actuators A Phys..

[28] Zhao P., Song Y., Xie P., Zhang F., Xie T., Liu G., Zhao J., Han S., Zhou Y. (2023). All-Organic Smart Textile Sensor for Deep-Learning-Assisted Multimodal Sensing (Adv. Funct. Mater. 30/2023). Adv. Funct. Mater..

[29] Mills J.A., Hamilton A.W., Gillespie D.I., Andonovic I., Michie C., Burnham K., Tachtatzis C. (2020). Identifying Defects in Aerospace Composite Sandwich Panels Using High-Definition Distributed Optical Fibre Sensors. Sensors.

[30] Mu Y., Liu Y., Dong Y., Liu J., Hou M., Sun Y., Li Y., Li J. (2026). Sustainable and multi-stimulus response coal-based carbon quantum dots hydrogel for wearable sensors. Polymer.

[31] Li L., Li Y., Ye Y., Guo R., Wang A., Zou G., Hou H., Ji X. (2021). Kilogram-scale synthesis and functionalization of carbon dots for superior electrochemical potassium storage. ACS Nano.

[32] Liu J., Yang S., Liu Q., Wang J., Mu Y., Li Y., Li J. (2024). Multi-stimulus response AIE fluorescent hydrogel for “Blind box” multistage secure information encryption. Chem. Eng. J..

[33] Liu Z., Chen L., Zhang X., Lu X., Peng M., Wang C., Liu Y., Zhang X. (2025). Carboxymethyl chitosan modified double-skeleton hydrogel electrolyte enables high performance for flexible zinc-air batteries. Int. J. Biol. Macromol..

